# A Randomized Controlled Trial of Qigong Exercise on Fatigue Symptoms, Functioning, and Telomerase Activity in Persons with Chronic Fatigue or Chronic Fatigue Syndrome

**DOI:** 10.1007/s12160-012-9381-6

**Published:** 2012-06-27

**Authors:** Rainbow T. H. Ho, Jessie S. M. Chan, Chong-Wen Wang, Benson W. M. Lau, Kwok Fai So, Li Ping Yuen, Jonathan S. T. Sham, Cecilia L. W. Chan

**Affiliations:** 1 Centre on Behavioral Health, The University of Hong Kong, Hong Kong, China; 2 Department of Anatomy, Li Ka Shing Faculty of Medicine, The University of Hong Kong, Hong Kong, China; 3 International Association for Health and Yangsheng, Hong Kong, China; 4 Department of Clinical Oncology, Li Ka Shing Faculty of Medicine, The University of Hong Kong, Hong Kong, China; 5 Department of Social Work and Social Administration, The University of Hong Kong, Hong Kong, China

**Keywords:** Qigong, Exercise, Chronic fatigue, Telomerase, Randomized controlled trial

## Abstract

**Background:**

Chronic fatigue is common in the general population. Complementary therapies are often used by patients with chronic fatigue or chronic fatigue syndrome to manage their symptoms.

**Purpose:**

This study aimed to assess the effect of a 4-month qigong intervention program among patients with chronic fatigue or chronic fatigue syndrome.

**Methods:**

Sixty-four participants were randomly assigned to either an intervention group or a wait list control group. Outcome measures included fatigue symptoms, physical functioning, mental functioning, and telomerase activity.

**Results:**

Fatigue symptoms and mental functioning were significantly improved in the qigong group compared to controls. Telomerase activity increased in the qigong group from 0.102 to 0.178 arbitrary units (*p* < 0.05). The change was statistically significant when compared to the control group (*p* < 0.05).

**Conclusion:**

Qigong exercise may be used as an alternative and complementary therapy or rehabilitative program for chronic fatigue and chronic fatigue syndrome.

## Introduction

Chronic fatigue is a common complaint in both primary care settings and in the general population. According to criteria established in the USA [1] and Canada [2], chronic fatigue is severe fatigue that persists or relapses for at least 6 months, while chronic fatigue syndrome, which is distinguished from chronic fatigue by severity and chronicity [3], is defined as “medically unexplained” fatigue lasting for six or more months. In recent years, researchers have paid increasing attention to chronic fatigue and chronic fatigue syndrome. The reported rates of chronic fatigue have ranged from 0.037 to 18.3 % in the general adult population [3–6]. The reported rates of chronic fatigue syndrome, however, have ranged between 0.007 and 2.8 % of the general adult population and between 0.006 and 3 % of patients seeking medical care [4, 7–9]. A population-based study in Hong Kong reported a point prevalence of chronic fatigue among adults as 10.7 % [3], while chronic fatigue syndrome was identified in 3 % of the local adult patients [10]. Chronic fatigue symptoms were reported as particularly common in women [6]. Given the unfavorable mental and physical outcomes of chronic fatigue and chronic fatigue syndrome that affect daily functioning, the burden of these conditions on healthcare and the economy should not be underestimated.

Though the etiopathogenesis of chronic fatigue syndrome is not yet well understood, the multifactorial disease pathways and the biopsychosocial model have been widely accepted as explanations for its complex symptoms and underlying mechanisms [11–13]. The inflammatory, immune, oxidative, and nitrosative pathways were suggested to induce a series of immunological and biological consequences that further impair physical and psychological functioning. According to this model, chronic fatigue symptoms develop through a developmental and cumulative process that involves different psychobiological factors contributing to triggering, predisposing, and maintaining stages. Among those factors, stress has been increasingly recognized as playing an important role in both the etiology and pathophysiology of chronic fatigue and chronic fatigue syndrome [14–16]. Clinically, psychiatric disorders are common among patients with chronic fatigue or chronic fatigue syndrome [3]. Research has demonstrated that both the occurrence of recent stressful life events and chronic stress levels appear to be increased in patients with chronic fatigue or chronic fatigue syndrome [17–20].

Because the pathophysiology of chronic fatigue and chronic fatigue syndrome remains inchoate, current treatment modalities mainly seek to alleviate symptoms [14]. To date, there are controversies regarding appropriate strategies for the treatment or management of chronic fatigue and chronic fatigue syndrome. Because western treatments and medications are often associated with limited clinical benefits [21] and possible undesirable side effects [22], complementary and alternative therapies are often used by individuals with chronic fatigue or chronic fatigue syndrome to manage their symptoms [23, 24]. From the perspective of traditional Chinese medicine, chronic fatigue and chronic fatigue syndrome are caused by blood stasis due to *qi* (vital energy) deficiency and/or emotional constrain; therefore, stimulation of the blood and *qi* circulation (Xing Qi Huo Xue, 行氣活血) is the core treatment strategy for chronic fatigue and chronic fatigue syndrome [22, 25].

Qigong, a mind–body exercise within the paradigm of traditional Chinese medicine, is practiced by a large number of people in Chinese communities. It aims to achieve a harmonious flow of energy (*qi*) in the body through gentle movements that integrate body, mind, and spirit, to improve physical fitness, overall well-being, and longevity. Several randomized controlled trials of qigong exercise have demonstrated health benefits for patients with chronic neck pain [26, 27], knee osteoarthritis [28], fibromyalgia [29], chronic obstructive pulmonary disease [30], or cancer [31]. Qigong exercise has also been applied in two pilot studies for the treatment of chronic fatigue and chronic fatigue syndrome [32, 33], and desirable effects on symptoms and physical and mental functioning have been suggested in these studies. The beneficial effects of qigong exercise for chronic fatigue and chronic fatigue syndrome should be further tested in large-scale randomized controlled trials.

To date, little is known about the effects of qigong exercise on telomeres and telomerase. Telomeres are protective DNA sequences at the ends of linear chromosomes that ensure chromosomal stability. They shorten with each cell division or under conditions of oxidative stress [34, 35]. Telomere length has been used as a “psychobiomarker” linking stress and disease [36]. Shortened telomere in humans is emerging as a marker of disease risk and progression, and premature mortality [37–39]. Telomere shortening can be counteracted by the cellular enzyme telomerase. Telomerase activity plays an essential role in cell survival by extending telomere length and protecting the chromosomes, which promotes cell growth and longevity. Previous studies have suggested that detectable changes in telomere length cannot be detected over a short period of time and at least 1 year may be needed [40]. Thus, testing telomerase activity is an optimal alternative and has been used in some studies. Previous studies have shown that both shortened telomere and lower telomerase activity are associated with chronic psychological distress such as chronic fatigue syndrome [40–43]. Recent findings suggest that telomerase activity may be improved by intensive meditation training [44], yogic meditation [45], physical exercise [46, 47], or comprehensive lifestyle changes [40]. However, little is known about whether telomerase activity can be improved by other forms of mind–body intervention, such as qigong exercise, which is considered a holistic health practice promoting physical and mental well-being as well as longevity.

Thus, the primary aim of this randomized controlled trial is to evaluate the effects of qigong exercise on fatigue-related symptoms and physical and mental functioning in patients with chronic fatigue or chronic fatigue syndrome. The secondary aim of this trial is to evaluate the effect of qigong exercise on telomerase activity, drawing on its potential impact on stress-related damage at the cellular level. We hypothesized that qigong exercise would lead to positive improvements in fatigue-related symptoms and functioning in persons with chronic fatigue or chronic fatigue syndrome and would result in increased telomerase activity.

## Methods

### Participants

Participants in this study were recruited from communities through advertising online and in local newspapers, as it is generally rare for patients with chronic fatigue or chronic fatigue syndrome alone to stay in public hospitals, and there is no register of or self-help organization for patients with chronic fatigue syndrome in Hong Kong. Eligible participants were adults aged 18 to 55 years (those older than 55 years were excluded due to high possibility of chronic illness) who were available at all testing points, were receptive to random allocation, and met the United States Centers for Disease Control (CDC) inclusion criteria for chronic fatigue syndrome [1], which are the most widely used in the field. According to these criteria, individuals with chronic fatigue or chronic fatigue syndrome are those with fatigue that has persisted or relapsed for six or more months and is accompanied by four or more of the following eight distinctive symptoms: impaired memory or concentration capacity, post-exertional malaise, sleep problems, muscle pain, arthralgia, headache, recurrent sore throat, and tender cervical or axillary lymph nodes. To minimize the impact of chronic illnesses as much as possible, those with any diagnosed medical conditions that might explain the presence of chronic fatigue were excluded. Examples of these conditions included cancer, hypothyroidism, sleep apnea, narcolepsy, hepatitis B or C virus infection, substance abuse, and mental disorders (e.g., major depressive disorder, bipolar affective disorders, schizophrenia), and severe obesity. We also excluded persons who had participated in qigong training within the previous 6 months and those with serious medical conditions that might limit their participation. All participants provided written informed consent. Ethical approval was obtained from the Institutional Review Board of the University of Hong Kong/Queen Mary Hospital/Hong Kong Hospital Authority West Cluster.

**Fig. 1:**
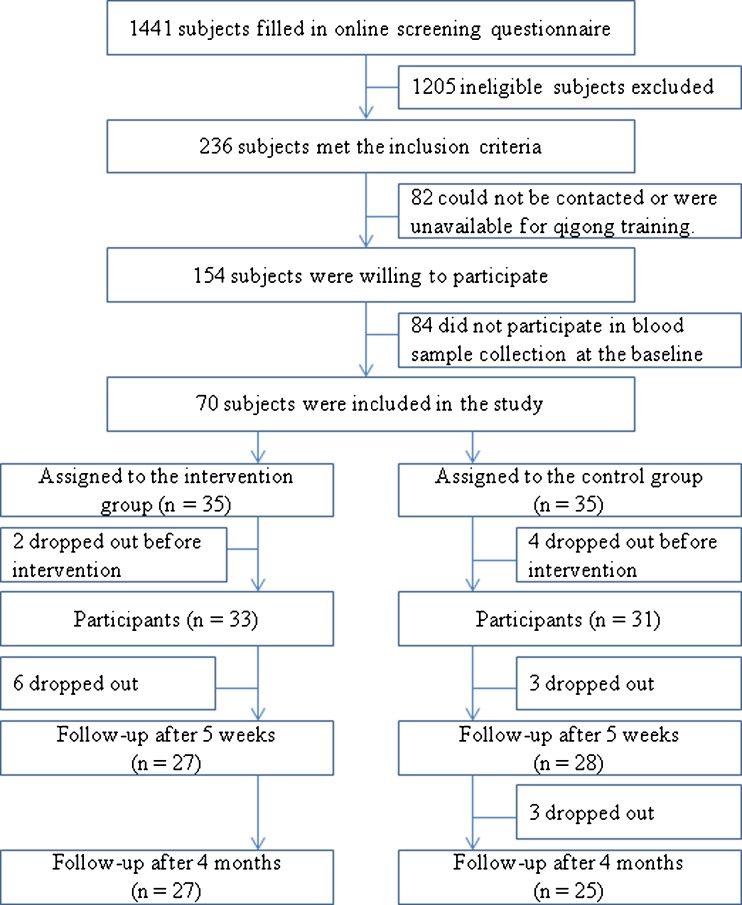
Flow chart of the selection of participants in the study

The study was advertised in the media, and 1,441 adults volunteered to fill in an online screening questionnaire. Two hundred and thirty-six subjects met the inclusion criterion, of which 82 subjects were excluded because they could not be contacted or were unavailable for the qigong training. Of the 154 subjects who were willing to take part in the study, 84 were excluded because they were unavailable for the blood sample collection at the baseline measurement (there was no significant difference in demographic characteristics and reported fatigue symptoms between this group of subjects and those included). Consequently, 70 subjects were included in this study, but 6 dropped out before qigong training (2 in the intervention group and 4 in the control group). Thus, the analyses were based on a final sample of 64 participants (33 in the intervention group and 31 in the control group). The recruitment target of 30 participants per group was calculated on the basis of a similar number of referred subjects in two previous studies on chronic fatigue syndrome [48, 49]. A trial with 60 participants would provide a power of 80 % to detect a 0.72 standardized difference at a two-sided significance level of 5 %. The flow chart of the selection of participants is presented in Fig. 1.

### Study Design and Procedure

This study was a randomized controlled clinical trial with repeated measures, which was conducted between October 2010 and March 2011. Each potential participant was required to complete an online screening questionnaire and was evaluated for eligibility by a pair of investigators with any discrepancies being resolved by discussion. Eligible participants were required to complete an additional questionnaire to measure the severity of their chronic fatigue symptoms and physical and mental functioning before intervention and at follow-ups. Following the online screening, the participants completed the baseline measures and gave a blood sample after having signed the written informed consent form. Each participant was assigned an order number before the baseline assessment. They were then randomly assigned to either the intervention group or a wait list control group according to the order numbers. For allocation of the participants, a computer-generated list of random numbers was used. Blinding the participants to the allocation was not possible due to the nature of intervention. The intervention program lasted 4 months, with group qigong training for 5 weeks followed by home-based qigong exercise for 12 weeks in the intervention group. Primary outcomes were self-perceived fatigue symptoms severity and physical and mental functioning, while secondary outcome was telomerase activity in peripheral blood mononuclear cells. Data for the subjective outcome measures were collected from both the intervention group and control group at three time points: pre-intervention (T0, baseline), post-training (T1, 5 weeks after), and post-intervention (T2, 4 months after). Blood samples from the participants were collected at two time points: pre-intervention (T0) and post-intervention (T2).

### Intervention

Participants assigned to the intervention group received group qigong exercise training (Wu Xing Ping Heng Gong, 五行平衡功) twice a week for five consecutive weeks, followed by home-based qigong exercise for 12 weeks. Each session of qigong exercise training lasted 2 h, with a brief introduction to the basic theories of traditional Chinese medicine or educational session on the physiology of mind–body connections (30–40 min), followed by mindful meditation for relaxation and mind concentration and then gentle movement or body stretching in standing postures to facilitate a harmonious flow of *qi* along the energy channels (20 min). Lastly, a 1-h session of qigong exercise training was delivered by an experienced Daoist qigong master (Yuen L.P.) with more than 20 years of experience in qigong practice as well as a background in traditional Chinese medicine. During the 4-month intervention program, all participants in the intervention group were required to engage in qigong exercise 30 min every day at home. To assess home exercise, they were required to report the frequency and duration as well as adverse effects of the self-practice at home at the end of the program. No adverse effects were reported. Participants assigned to the control group were advised to undertake normal activities but were asked to refrain from joining any outside qigong training class. No participants in the control group joined an outside qigong class as qigong exercise training was provided to them after the final outcome measurement.

### Measures

#### Screening Measures

Each potential participant was required to complete an online screening questionnaire that elicited the following: (1) demographic information consisting of age, gender, employment status, education level, marital status, religion, and the number of family members living together with the participant; (2) an item to indicate whether or not fatigue symptoms persisted or relapsed for six or more months, and a list of the eight distinctive chronic fatigue symptoms recommended in the CDC inclusion criteria for chronic fatigue syndrome [1]; (3) a checklist of medical history based on the CDC exclusion criteria for chronic fatigue syndrome [1]; and (4) reported lifestyle in the previous 6 months, including details on exercise, smoking, and alcohol consumption.

#### Chalder’s Fatigue Scale

The severity of chronic fatigue was assessed by using Chalder’s Fatigue Scale [50], which was developed to measure the severity of fatigue over the previous 6 months. The scale consists of 14 items for evaluating two dimensions of chronic fatigue: physical fatigue (8 items) and mental fatigue (6 items) on a five-point Likert scale (none, better than usual, no more than usual, worse than usual, much worse than usual). The five responses are scored from 0–4. The subscale scores are equal to the summed scores for the physical fatigue and mental fatigue items. The total fatigue score is obtained by summing all of the items [50]. The scale was found to be both reliable (*r* = 0.86 for physical fatigue, and *r* = 0.85 for mental fatigue) and valid (Cronbach's *α* = 0.89). A recent study of the Chinese version of Chalder’s Fatigue Scale also supported the notion of a two-factor structure in the community sample, and the internal consistency of the Chinese version was good (*α* = 0.863) [51].

#### Medical Outcomes Study 12-Item Short-Form Health Survey

Physical functioning and mental functioning were assessed by the Chinese version of the Medical Outcomes Study 12-Item Short-Form Health Survey [52, 53]. The 12 items are classified into two subscales to measure the two dimensions of health: physical functioning (6 items) and mental functioning (6 items). Accordingly, two summary scores are yielded. Each subscale is scored on a 0–100 scale in which 100 indicates the best possible score and 0 indicates the worst.

#### Measurement of Telomerase Activity

For each participant, 3 ml of peripheral blood was collected in heparinized tubes. The peripheral blood mononuclear cells were isolated from each blood sample using Ficoll-Paque PLUS (GE Healthcare). In brief, 1 ml of blood sample was layered on 750 μl of Ficoll-Paque, and subsequently centrifuged at 400×*g* for 20 min at room temperature. The upper plasma layer was then removed, and the monocyte layer was collected immediately following centrifugation. The extracts were stored at −80 °C until further processing. Telomerase activity was tested by a commercially available kit TeloTAGGG telomerase PCR ELISA (Roche) according to the protocol. In brief, 10 μl of mononuclear cells isolated were lysed in 200 μl of lysis buffer, and 3 μl of the obtained protein extract was used for the subsequent telomerase-catalyzed primer elongation and PCR reaction. Biotin-labeled primers were used in the PCR reaction, then 5 μl of the PCR product was denatured, hybridized to digoxigenin-labeled probes, and immobilized to streptavidin-coated wells. Finally, the digoxigenin-labeled products were visualized by a peroxidase-conjugated anti-digoxigenin antibody and tetramethyl benzidine acting as a peroxidase substrate. The level of telomerase activity is in proportion to the colorimetric measures. The values were normalized to the positive control provided in the ELISA kit, which is in a relative manner. For each participant, telomerase activity in the peripheral blood mononuclear cells was assayed at pre-intervention and at the end of the 4-month intervention. The blood samples were collected in an identical manner, at the same time of day, and at the same location, and were treated identically at all steps. The group allocation was blinded for laboratory technicians.

### Statistical Analysis

Continuous data were summarized by means and standard deviations, and categorical data were summarized by frequency. Differences at baseline for the demographic information, lifestyles, and reported fatigue symptoms between the two groups were compared using a chi-squared test for categorical data and a *t* test or pairwise *t* test for continuous data. The intragroup differences between different time points for each outcome measure were compared using a pairwise *t* test. Cohen’s *d* statistic was also calculated to determine effect size for each outcome. Repeated measures analyses of covariance (ANCOVA) were then performed to test the interaction effect of time and group for each outcome, adjusting for two fatigue symptoms (muscle pain, and tender cervical or axillary lymph nodes). Effects were evaluated on an intention-to-treat basis, and the missing values were substituted by way of mean imputation for each outcome measure [54]. All data analysis was conducted with SPSS (version 18.0, SPSS Inc., Chicago, IL). A *p* value less than 0.05 was considered as statistically significant.

## Results

### Demographic Characteristics and Lifestyles at Baseline

Table 1 shows the baseline data for the demographic characteristics, lifestyles, and self-reported chronic fatigue symptoms of the intervention group and the control group. The mean ages were 42.1 years (range 23–52) in the intervention group and 42.5 years (range 29–51) in the control group, respectively. More than three quarters of participants were female (75.8 and 83.9 % in the two groups, respectively). As shown in the table, baseline characteristics were reasonably well balanced between the two groups. There were no significant differences in lifestyles and the number of chronic fatigue symptoms between the two groups, apart from a just significant intergroup difference in the two types of fatigue symptoms (muscle pain, and tender cervical or axillary lymph nodes, *p* < 0.05).

**Table 1:** Baseline characteristics, lifestyles, and chronic symptoms

Demographic	Intervention (*n* = 33)		Control (*n* = 31)		*p* ^a^
	Mean (SD)	*N* (%)	Mean (SD)	*N* (%)	
Age (years)	42.1 (7.3)		42.5 (5.5)		0.849
Gender					
Female		25 (75.8 %)		26 (83.9 %)	0.420
Education					0.326
Secondary or high school		13 (39.4 %)		16 (51.6 %)	
College or above		20 (60.6 %)		15 (48.4 %)	
Marital status					0.756
Unmarried		9 (27.3 %)		11 (35.5 %)	
Married/cohabited		21 (63.6 %)		17 (54.8 %)	
Divorced/separated		3 (9.1 %)		3 (9.7 %)	
Employment					0.374
Full time		27 (81.8 %)		24 (77.4 %)	
Part time		2 (6.1 %)		0	
Housewife		3 (9.1 %)		6 (19.4 %)	
Unemployed		1 (3.0 %)		1 (3.2 %)	
Religion involvement					
Yes		8 (24.2 %)		12 (38.7 %)	0.212
Number of family members living together					0.610
None		4 (12.1 %)		3 (9.7 %)	
1–2		14 (42.4 %)		17 (54.8 %)	
3 or more		15 (45.5 %)		11 (35.5 %)	
Lifestyles					
Doing exercise regularly		9 (27.3 %)		3 (9.7 %)	0.071
Smoking		1 (3.0 %)		2 (6.5 %)	0.518
Alcohol drinking		12 (36.4 %)		11 (35.5 %)	0.942
Fatigue symptoms (at least 6 months)					
Impaired memory or concentration capacity		31 (93.9 %)		29 (93.5 %)	1.000
Post-exertional malaise		29 (87.9 %)		29 (93.5 %)	0.673
Sleep problems		32 (97.0 %)		30 (96.8 %)	1.000
Muscle pain		27 (81.8 %)		31 (100 %)	0.025
Arthralgia		24 (72.7 %)		23 (74.2 %)	0.894
Headache		21 (63.6 %)		23 (74.2 %)	0.362
Recurrent sore throat		18 (54.5 %)		17 (54.8 %)	0.981
Tender cervical or axillary lymph nodes		19 (57.6 %)		26 (83.9 %)	0.021
Average number of reported fatigue symptoms	6.1 (1.4)		6.7 (1.3)		0.072

^a^Chi-squared test for categorical variables and *t* test for continuous variables

Twenty-seven participants (81.8 %) in the intervention group and 25 (80.6 %) in the control group completed the 4-month program. Six participants in each group withdrew from the study (Fig. 1). Three additional participants in the intervention group and one additional participant in the control group did not fill in the questionnaire but provided a blood sample for telomerase activity testing at the end of the intervention program.

### The Efficacy of Intervention

Table 2 shows the intragroup and intergroup differences of the subjective outcome measures, including the total fatigue score, physical fatigue score, mental fatigue score, physical functioning score, and mental functioning score. Compared to baseline values, the total fatigue score (*p* < 0.001, *d* = −1.5), physical fatigue score (*p* < 0.001, *d* = −1.9), mental fatigue score (*p* = 0.001, *d* = −0.9), and mental functioning score (*p* < 0.001, *d* = 1.3) were significantly improved in the intervention group at 5 weeks, and the improvement was maintained at 4 months. Only the physical functioning score was not significantly changed in the intervention group. In the control group, the total fatigue score, physical fatigue score, and mental fatigue score were also significantly improved at 5 weeks (*p* = 0.003, *d* = −0.7; *p* = 0.001, *d* = −0.6; and *p* = 0.031, *d* = −0.6 respectively) and at the end of the 4-month program (*p* = 0.001, *d* = −1.0; *p* = 0.001, *d* = −0.9; and *p* < 0.001, *d* = −0.9 respectively). The physical functioning score and the mental functioning score were not significantly improved in the control group compared to baseline values.

**Table 2:** Intragroup and intergroup comparisons for subjective outcomes using repeated measures ANCOVA adjusting for fatigue symptoms (muscle pain, tender cervical or axillary lymph nodes) at baseline

	Baseline (T0)	5 weeks after (T1)^a^		4 months after (T2)^a^		Time × group effect	
	Mean (SD)	Mean (SD)	Effect size (*d*)	Mean (SD)	Effect size (*d*)	*F*	*p*
Total fatigue score							
Intervention group	39.9 (6.3)	26.3 (10.9)***	−1.5	21.6 (10.4)***	−2.1	12.93	0.000
Control group	39.7 (6.1)	34.8 (8.0)**	−0.7	32.1 (8.8)***	−1.0		
Physical fatigue score							
Intervention group	25.0 (3.7)	15.2 (6.5)***	−1.9	12.9 (6.1)***	−2.4	20.09	0.000
Control group	24.7 (4.1)	21.8 (5.1)**	−0.6	20.3 (5.7)***	−0.9		
Mental fatigue score							
Intervention group	14.9 (3.6)	11.1 (5.1)**	−0.9	8.8 (4.6)***	−1.5	4.60	0.012
Control group	15.0 (3.2)	13.0 (3.9)*	−0.6	11.9 (3.8)**	−0.9		
Physical functioning score							
Intervention group	36.9 (7.2)	38.4 (6.1)	0.2	40.1 (6.9)	0.5	0.69	0.484
Control group	35.7 (7.1)	37.5 (8.1)	0.2	37.8 (5.6)	0.3		
Mental functioning score							
Intervention group	32.5 (10.7)	43.8 (6.9)**	1.3	42.7 (7.2)**	1.1	7.60	0.001
Control group	33.5 (9.6)	34.6 (9.6)	0.1	35.7 (9.5)	0.2		

*p < 0.05; **p < 0.01; ***p < .0.001

^a^Compared with baseline values

As shown in Table 2, the difference in the change of each outcome measure was examined with the time and group interaction effect. Compared to the control group, the improvement in the total fatigue score [*F*(2, 120) = 12.93, *p* < 0.05], physical fatigue score [*F*(2, 120) = 20.09, *p* < 0.01], mental fatigue score [*F*(2, 120) = 4.60, *p* < 0.05], and mental functioning score [*F*(2, 120) = 7.60, *p* = 0.001] was significant in the intervention group, whereas the changes in the physical functioning score in the intervention group were not significant [*F*(2, 120) = 0.69, *p* > 0.05].

In Table 3, the telomerase activity at baseline and at the end of the 4-month intervention program was compared within and between the two groups. Compared to baseline values, telomerase activity was significantly improved at the end of the program in the intervention group (*p* = 0.033, *d* = 0.52), but was not improved in the control group. The change of telomerase activity in the intervention group was statistically significant when compared to the control group, as indicated by the interaction effect of time and group [*F*(2, 120) = 5.03, *p* < 0.05].

**Table 3:** Intragroup and intergroup comparisons for telomerase activity using repeated measures ANCOVA adjusting for fatigue symptoms (muscle pain, tender cervical, or axillary lymph nodes) at baseline

Telomerase activity (arbitrary unit)	Baseline (T0)	4 months after (T2)		Time × group effect	
	Mean (SD)	Mean (SD)	Effect size (*d*)	*F*	*p*
Intervention group	0.102 (0.051)	0.178 (0.201)*	0.52	5.03	0.029
Control group	0.089 (0.036)	0.104 (0.059)	0.31		

*p < 0.05

## Discussion

The results of the current study suggested that qigong exercise improved fatigue symptoms for patients with chronic fatigue or chronic fatigue syndrome, as indicated by the significant change in the total fatigue score. This result confirms the findings of an uncontrolled pilot study [32] and a small-scale randomized controlled trial of qigong exercise among patients with chronic fatigue syndrome [33]. Our results further indicated that qigong exercise improved physical fatigue symptoms more significantly than mental fatigue symptoms. Given the nature of chronic fatigue syndrome, these effects are understandable. Clinically, chronic fatigue syndrome is also named myalgic encephalomyelitis. Diagnosed chronic fatigue syndrome is actually a neurological disorder, rather than a physical or mental problem alone. Patients with chronic fatigue syndrome may be subject to cognitive deterioration or impairment [55]. The mental fatigue subscale of the Chalder’s Fatigue Scale includes items to measure such dimensions as memory, concentration, problem thinking, and difficulty finding correct words. These dimensions are generally related to cognitive functioning. Generally, it is more difficult to improve cognitive function than psychological well-being or mental health, including such dimensions as energy and fatigue, body pain, and psychological wellness as measured by the SF-12.

In the current study, the mental functioning score in the qigong group was significantly improved compared to controls. In a prior pilot randomized controlled trial on chronic fatigue syndrome [33], bodily pain and mental health as measured by SF-36 were significantly improved in the qigong group compared to the baseline values, but the difference in changes between the qigong group and the control group was not significant, possibly due to the study’s small sample size (*n* = 31). Our study indicated that physical functioning measured by the physical health subscale of SF-12 was not significantly improved in the qigong group when compared to the controls. These results are inconsistent with previous findings in an uncontrolled study of qigong over 6 months among patients with chronic fatigue syndrome [32], in which both physical functioning and mental health as measured by the 116-item RAND Medical Outcomes Study questionnaire were significantly improved. Our results suggest that short-term qigong exercise may improve fatigue symptoms but is not as effective in improving physical functioning for patients with chronic fatigue or chronic fatigue syndrome. The improvement in physical functioning may instead require long-term qigong exercise.

The current study is the first randomized controlled clinical trial to measure the effect of qigong exercise on telomerase activity for individuals with chronic fatigue or chronic fatigue syndrome. Findings from this study indicated that telomerase activity was significantly improved among patients with chronic fatigue or chronic fatigue syndrome who received qigong exercise intervention over a 4-month period, and the change in telomerase activity in the qigong group was statistically significant when compared to the control group. This result confirms the findings in previous studies on intensive meditative training [44], yogic meditation [45], and comprehensive lifestyle changes [40]. In a pilot study among 30 adult patients with prostate cancer, Ornish et al. [40] reported that 3 months of intensive lifestyle changes increased telomerase activity in peripheral blood mononuclear cells; however, there was not a usual care control group in the study. In a recent randomized controlled trial [44] of intensive meditation training among 60 healthy subjects, 46 (20 meditation retreat participants and 26 controls) participated in telomerase activity assessment and the results suggested greater telomerase activity in the retreat participants than in the control group after 3 months of intensive meditation training, but there was no baseline measure of telomerase activity in the study. Therefore, generalization of the findings from these two studies is limited. Most recently, Lavretsky and colleagues [45] conducted a pilot randomized controlled trial to examine the effect of 8 weeks of brief daily yogic meditation on immune cell telomerase activity in family dementia caregivers with mild depressive symptoms, and the results indicated a marginally significant difference (*p* = 0.05) in the change of telomerase activity between the meditation group (*n* = 23) and the relaxation control group (*n* = 16), probably due to small size in the study. In the current randomized trial on the basis of a justified sample of patients with chronic fatigue or chronic fatigue syndrome, we examined the effect by comparing the intervention group with the control group and adjusting for the baseline values. The result suggested a statistically significant association between qigong exercise and cellular telomerase activity.

As mentioned before, chronic fatigue and chronic fatigue syndrome are often associated with a high level of perceived stress and psychiatric disorders including depression, which are common among patients with chronic fatigue or chronic fatigue syndrome. Although a recent study [56] has suggested increased telomerase activity in major depression, explanations of the finding remain speculative and a number of studies have suggested low telomerase activity in response to psychological and oxidative stress as experienced by patients with chronic fatigue or chronic fatigue syndrome [43, 57, 58]. It is suggested that low telomerase activity could result in shortened telomeres by limiting the replenishment of impaired telomeres [59]. The underlying mechanism for the beneficial effects of qigong exercise as a mind–body intervention on telomerase activity in the current study may be explained in terms of two major components of qigong exercise. During qigong exercise, mindfulness, a key component of intensive meditative training, is often effectively cultivated. Thus, qigong exercise is often used as a stress management strategy and is potentially effective in enhancing cellular telomerase activity for patients with chronic fatigue or chronic fatigue syndrome through the reduction of the oxidative stress level [43, 60] and regulating immune response [43, 44]. The hypothalamic–pituitary–adrenal axis is an important pathway for these effects [61]. Another underlying mechanism may be that qigong exercise as a low-impact physical activity may increase serum concentrations of insulin-like growth factor (IGF) 1, which enhances telomerase activity and delays cellular aging [46]. Even though a retrospective study [62] among 69 healthy adults aged 50–70 years did not observe a significant association between physical activity level and telomerase activity, a study in mice indicated that short-term physical exercise (21 days) upregulated telomerase activity to more than twice that of sedentary controls [46]. Another study also indicated that physical exercise increased telomerase activity and neurogenesis in a mouse model of schizophrenia [47].

Although the results of this study are promising, the study has some limitations. One of major limitations is that participants did not receive a medical examination from a physician at the beginning of the study, so some of them might have not fully met the CDC criteria for chronic fatigue syndrome. Thus, the effects on telomerase activity and subjective outcomes might have been confounded by possible chronic disorders. Further studies should strictly adhere to the CDC diagnostic criteria for chronic fatigue syndrome. Another limitation is that the current study was neither double blind nor included a control group that did something equivalent to the controls, such as health education class. Thus, the effects might be due to demand characteristics, the Hawthorn effect, or possible placebo effect of being in a group. Moreover, telomerase activity was not tested during post-training; thus, attributing increased telomerase activity to qigong may be challenged given the unknown reaction time for changing telomerase activity after intervention. Furthermore, it is possible that other factors besides qigong exercise such as meditation, physical activity, body weight and life style change including diet, and inflammation or infections [40, 43–47] might have contributed to the increase in telomerase activity over a period of 4 months. These confounders should be strictly controlled in future studies. Finally, participants’ stress levels were not measured before and after the invention, so the mediating effect of decreased psychological stress in the link between qigong exercise and the change in telomerase activity could not be examined. Due to aforementioned limitations, interpretation and generalization of the association between qigong exercise and telomerase activity observed in this study should be cautious. This finding needs to be replicated in further carefully designed randomized controlled trails, but it does raise the possibility of the involvement of telomerase as an important biomarker for mind–body interventions.

## Conclusion

In conclusion, the results of the present study indicate that qigong exercise may improve chronic fatigue symptoms and mental functioning and result in increased telomerase activity for patients with chronic fatigue or chronic fatigue syndrome. The improvement in telomerase activity may be an alternative pathway of the potential health benefits of qigong exercise as a mind–body intervention. The results suggest that qigong exercise as a modality of mind–body therapy may be used as an alternative and complementary therapy or rehabilitation program for chronic fatigue and chronic fatigue syndrome.
